# Cæsarean Section

**Published:** 1856-08

**Authors:** Charles S. Mills


*Cæsarean Section.* BY CHARLES S. MILLS, M. D.

On Monday, the 12th of May, 1856, between eight and nine o'clock in the evening, I was requested to attend immediately a negro woman belonging to Mr. Thomas Samanni, of this city, in labor with her first child. My friend, Dr. Beale, the family physician of Mr. S., was prevented, by severe indisposition, from attending, and the case was entrusted to my care. At an early period, he had recognized the pregnancy and the difficulties by which the delivery of a child at maturity would be attended, and for the safety of the mother, had recommended a premature delivery. His advice having been rejected, he looked forward with anxiety to her approaching labor, and had spoken freely to me upon the subject. On entering the room, therefore, I was not surprised to recognize in the person of my patient, a little creature well known in our city on account of her diminutive size. She is twenty-three years of age, has generally enjoyed good health, has a large and well developed head, chest broad and full, pelvis narrow, with limbs and body remarkably short, her height being only three feet nine inches. I found her walking about the room apparently in great pain. On inquiry, I learned that she had been complaining since the previous evening, having passed a restless night, but that the violence of the pains had greatly increased within two or three hours; she was supposed to be at full term of gestation. Causing her to be placed in position for an examination, the finger readily passed along the vagina to the os uteri, which was soft and sufficiently open to admit its extremity, and felt through the membranes what I at first thought was an arm of the child lying beside its head. A more careful examination, however, soon satisfied me that instead of the child's head, this was the promontory of the sacrum projecting across the pelvis above the pubis, and leaving a space between the two bones scarcely wide enough to allow two fingers to pass, while just above the symphisis and apparently on the pubis was either an arm or a leg but which I could not yet determine. Recognizing at once the utter impossibility of effecting delivery without the aid of instruments, I left the patient with her female attendants, ordering an enema for her, and proceeded to my office to procure them. Returning in about an hour, and finding the pains active with the os uteri dilatable, I ruptured the membranes and endeavoured to pass my hand up, determined to bring down the foot if a leg presented, or turn the child in case the presenting part should be an arm. As the waters escaped I made an effort to pass my hand through the superior strait, but succeeded only in passing two fingers, and at the same moment feeling a foot of the child I brought it down. I then endeavored to find its fellow, but being unable to reach that or any other portion of the child, I tried, by traction on the presenting leg, which I now recognized as the right, with its instep turned to the hollow of the sacrum, to force down the body into the pelvis. After using as much force as I thought judicious, and finding that the body still remained out of reach, I determined to send for assistance. At least an hour elapsed before the arrival of Dr. Deane, who was absent when my messenger reached his house. Explaining to him what had been done by me, I expressed the opinion that the deformity was so great as to render embriulcia impossible, and that we should be compelled to resort to the cæsarian section. The Doctor then made a very careful and patient examination, endeavoring to bring down the body of the child within reach of the instruments for embryotomy; but though very powerful traction was applied to the presenting leg, which seemed to fill the space between the symphisis pubis and the promontory of the sacrum, the body of the child remained immovably fixed above the entrance to the superior strait, and out of reach. He then concurred with me in the opinion that embriulcia was impracticable, and that the only chance of delivery was in the cæsarian section. Not having with me the instruments and apparatus requisite for the performance of that operation, I left him with the patient, and started to procure them from my office, when I met at the front door of the house, Dr. Bolton, who had likewise been sent for. I requested him to join Dr. Deane whilst I proceeded to get the instruments. Soon after my return Dr. Drew (whom I was happy to learn had been sent for by Dr. Deane, and, but for the lateness of the hour, near midnight, I should have requested other medical friends to lend me their encouragement in so grave an operation,) entered the room and was asked to examine the case and confer with us.

It was now proposed that the patient should be anæthetised, and an effort made to reach the abdomen of the child in order to eviscerate it, if after a more thorough examination, it should appear that the child could then be brought away. This was accordingly done, and Dr. Bolton with great difficulty succeeded in passing two fingers through the superior strait so as to reach with their extremities the abdomen of the child, but could make no use of them to conduct an instrument with certainty or safety to the mother, and was of opinion that it would be impossible to deliver the child through so narrow a passage even if we could succeed in eviscerating it. Being still loath to resort to the cæsarian section, until every effort to deliver per vias naturales had been tried and failed, the presenting leg was now enveloped in a bandage, and, the mother being still under the influence of chloroform, gradual but very powerful traction was made, hoping still to force down the body into the pelvis. The greatest force which could be applied, without risking the laceration and separation of the limb, produced no other effect than to bring down the thigh a little lower, which was so firmly bound between the pelvis and the promontory of the sacrum as to cause strangulation of the leg and foot. Upon consultation it was now unanimously thought that the cæsarian section should be made without farther delay, and accordingly, a table was prepared in the centre of the room and the requisite arrangements made for the operation. The patient was again brought fully under the influence of chloroform, and removed from the bed to the table, her female attendants being excluded from the room.

*Operation.*—Catheterism having been effected, though with some difficulty, in consequence of the interference of the fœtal leg and the displacement of the bladder, the patient lying on her back, with her head and shoulders slightly raised on pillows, and her knees flexed over the edge of the table, I stationed myself as most convenient, on her left side, and made an incision through the integuments from about two inches above the symphisis pubis along the line of the linea alba, passing around the umbilicus to about an inch above. The abdominal walls, being thin, were cautiously dissected along the length of the first incision down to the peritoneum, which was carefully incised so as to admit the point of the finger to serve as a director, and the incision extended upwards and downwards, giving exit to a large quantity of serum, and laying bare the body of the uterus with a small knuckle of intestine overlapping its fundus, while the contracted bladder lay at the lower terminus of the incision and covering the cervix. The contact of cool air seemed to stimulate the uterus to contraction, and as it was no longer kept in place by the abdominal walls, I spread my hands over it to sustain it until it became quiescent. So far there had been no hemorrhage, and as the patient was fully anæthetised, (beautifully exemplifying the happy effects of chloroform) there was no necessity for holding her, and the abdominal viscera remained throughout the operation quietly in position, and offered no impediment whatever. A vertical incision was now cautiously made throughout the walls of the uterus, corresponding in direction with the external one, and about 6 or 7 inches long, dividing in its course a large uterine sinus, followed by a gush of blood, and exposing the left shoulder and arm of the fœtus, which I seized with my left hand and raised the child, while introducing my right, I caught hold of its neck and flexing it on the body, readily extracted it from the womb, which immediately commenced contracting. The placenta was detached and came away with the fœtus, of whose vitality I was assured by the pulsation felt in the vessels of the neck. The child was handed to one of the gentlemen present, whilst I, twisting the membranes around the placenta, withdrew them from the uterus. Dr. Bolton undertook the resuscitation of the child, which, in a few minutes gave utterance to the usual cry so grateful to the ears of the anxious attendants, and in the meantime my hand was kept upon the uterus stimulating it to contraction, and excluding from between its incised walls any viscus which might offer to enter its cavity. The womb being now firmly contracted and the hemorrhage checked, the clots of blood were carefully removed, and the edges of the external wound brought together and secured with six or eight interrupted stitches and a broad circular bandage. The patient was now replaced in bed and made as comfortable as possible. She had now recovered her consciousness, her pulse being very good, and general condition as favorable as could be desired, when we left her with her female attendants, enjoining perfect quiet, and ordering that a pill containing a grain of opium should be given to her every hour, until sleep was produced.

Tuesday, 13th, 9, A. M.—Patient has taken three of the opium pills; slept some and feels comfortable; pulse 104; soft and compressible; abdomen tender and slightly tympanitic; skin moist and cool; tongue clean, and countenance good; applied four or five strips of adhesive plaster to sustain the stitches; covered the wound with a compress, and covered the abdomen on each side with a warm poultice; ordered the opium to be continued, and ice to be given when wanted.

12, M.—Complains of the afterpains and of soreness along the line of the incision, which, however, have not prevented her sleeping; has taken two of the pills since the morning's visit. Opium continued.

4, P. M.—Is in less pain; has slept well, and passed urine without assistance; some oozing observed from the lower edge of the incision. Renewed the poultice.

10, P. M.—Feels comfortable; pulse 108; no headache, tongue clean and moist; countenance good; has taken altogether six grains of opium; has no desire for food; thirst diminished; give the pills only when in severe pain.

On the following day the same treatment was continued. In the evening, pain, fever, and cough being present, the patient was bled with much relief. On the 15th, morphia and poultices continued. On the 16th, patient allowed half a pint of buttermilk in small quantities—no morphia through the day—repeated at night. On the 17th, she took a little boiled rice and milk; morphia repeated at bed-time. On the 18th, removed stitches and gave her 15 grs. of calomel. On the 19th, an enema with happy effect; no food through the day. From this time forward, the patient favorably progressed in ever respect, even to nursing her child. On Monday the 26th, the following report is given of her condition.

Monday, 26th.—Did not rest very well last night, suffering from distention of the bowels; tongue, however, clean and moist; pulse 96 and soft; skin cool and countenance cheerful. An ounce of castor oil was administered, which purged freely during the day with relief to all her unpleasant symptoms.

From this time, nothing in her condition occurred worthy of note until the 30th of May, when she complained of pain in the calf of the left leg, which I found tender on pressure, the tenderness circumscribed and unaccompanied by hardness or swelling. There was no febrile action, but tracing the course of the femoral vessels, no pain was felt until the hand approached the ramus of the pubis where the vessel could be felt hard beneath the fingers and painful. There was also pain felt in the iliac fossa when pressed on. As the bowels were still torpid, I ordered ten grains of calomel at night; which dose not purging, was repeated in the morning and followed by an ounce of castor oil at night. At the same time, the leg was enveloped in a warm poultice; the medicines purged freely, and the poultices were renewed night and morning, under which treatment the tenderness gradually disappeared after several days, leaving, however, a feeling of stiffness in the limb and a sense of drawing when an effort is made to extend it or put the foot to the ground.

In consequence of this phlebitis, she has not been allowed to leave her bed, though she is in other respects sufficiently well to do so. The child has not had a moment's sickness since its birth, and no medicament whatever, has ever been given to it. It is a fine hearty child and rather above the average size of children.

*Monthly Stethescope and Medical Reporter, (Richmond, Va.)*

